# Antioxidant Activity of *Lawsonia inermis* Extracts Inhibits Chromium(VI)-Induced Cellular and DNA Toxicity

**DOI:** 10.1093/ecam/nep205

**Published:** 2011-06-20

**Authors:** Gunjan Guha, V. Rajkumar, R. Ashok Kumar, Lazar Mathew

**Affiliations:** School of Biotechnology, Chemical and Biomedical Engineering, VIT University, Vellore 632 014, India

## Abstract

Hexavalent chromium Cr(VI) is a very strong oxidant which consequently causes high cytotoxicity through oxidative stress. Prevention of Cr(VI)-induced cellular damage has been sought in this study in aqueous and methanolic extracts of *Lawsonia inermis* Linn. (Lythraceae), commonly known as *Henna*. The extracts showed significant (*P* < .05) potential in scavenging free radicals (DPPH^•^ and ABTS^•+^) and Fe^3+^, and in inhibiting lipid peroxidation. DNA damage caused by exposure of pBR322 to Cr(VI)-UV is markedly inhibited by both extracts in varying degrees. A distinct decline in Cr(VI)-induced cytotoxicity was noticed in MDA-MB-435S (human breast carcinoma) cells with an increase in dosage of both extracts individually. Furthermore, both extracts proved to contain a high content of phenolic compounds which were found to have a strong and significant (*P* < .05) positive correlation to the radical scavenging potential, lipid peroxidation inhibition capacity and cyto-protective efficiency against Cr(VI)-induced oxidative cellular damage. HPLC analysis identified some of the major phenolic compounds in both extracts, which might be responsible for the antioxidant potential and the properties of DNA and cyto-protection. This study contributes to the search for natural resources that might yield potent therapeutic drugs against Cr(VI)-induced oxidative cell damage.

## 1. Introduction

Hexavalent chromium [Cr(VI)] is the most toxic and mutagenic heavy metal in biological systems [1]. It exists as oxo-species such as CrO_3_ and CrO_4_^2−^, which are robustly oxidizing [2], leading to excessive cytotoxicity that in turn may cause dermal damage, gastrointestinal bleeding, renal failure, intravascular hemolysis, liver damage, coma and even death [3]. Cr(VI) is transported into cells through the sulfate transporter [4], and leads to alteration of signal transduction pathways [5], cell transformation [6], and increases the risk for developing cancer [7]. Simultaneously, it is also known to inhibit cell proliferation/cell cycle [8], thereby inducing growth arrest, accompanied by the generation of reactive oxygen species (ROS) that presumably triggers oxidative damage to DNA [9] and consequent apoptosis [10]. Oxidative damage is associated with the generation of free radicals in cells exposed to Cr(VI) ion, and a propensity of cells to develop mutations in response to Cr(VI)-induced oxidative damage has been reported [11, 12].

Sources of Cr(VI) toxicity are broadly classified into occupational and non-occupational exposure types. Highest occupational exposures to Cr(VI) occur during chromate production, welding, chrome pigment manufacture, chrome plating and spray painting. Non-occupational sources of exposure include food, air and water [7].

Various compounds with differential antioxidant properties are found in floral resources which are considered to have high potential in context of therapeutic approaches to encounter and prevent free radical damage as that caused by Cr(VI) toxicity. *Lawsonia inermis* Linn. (Lythraceae), commonly known as *Henna*, is a popular skin and hair coloring agent in many parts of the world. In addition, it is traditionally used as a medicinal plant [13] by diverse groups of tribal/ethnic people [14–16]. *Lawsonia inermis* is used as an antirheumatic and antineuralgic agent [15], and also has potential as an antidiabetic drug [16]. There is evidence of the plant having wound healing properties [17]. Furthermore, treatment with hydroalcoholic extract of *L. inermis* (*in vivo*) has been proved to increase levels of cellular antioxidant enzymes such as glutathione reductase, superoxide dismutase and catalase [18].

This study was aimed at evaluating the effects of aqueous and methanolic extracts of *L. inermis* on induced oxidative toxicity in MDA-MB-435S (human breast carcinoma) cells, along with an estimation of the compositions of these extracts and their respective antioxidant potential.

## 2. Methods

### 2.1. Chemicals and Reagents

Chromium trioxide (CrO_3_), thiobarbituric acid (TBA), phenazine methosulfate (PMS) (also known as *N*-methylphenazonium methosulfate), L-15 (Leibovitz) cell culture medium (with l-glutamine), 2,2-diphenyl-1-picrylhydrazyl (DPPH), Dulbecco's phosphate buffered saline (PBS) (Ca^2+^/Mg^2+^-free) and 2,4,6-tri- pyridyl-*s*-triazine (TPTZ) were purchased from Himedia Laboratories Pvt Ltd (India). Trolox (6-hydroxy-2,5,7,8-tetramethyl chroman-2-carboxylic acid) and 2,2′-azino-*bis*(3-ethylbenz-thiazoline-6-sulfonic acid) (ABTS) were procured from Sigma Aldrich Chemical Co. (Milwaukee, WI, USA). MDA-MB-435S cell line was obtained from National Center for Cell Science (Pune, India). XTT {2,3-*bis*(2-methoxy-4-nitro-5-sulfophenyl)-5-[(phenylamino) carbonyl]-2H-tetrazolium hydroxide} was obtained from Sigma Chemical Co. (St Louis, MO, USA). pBR322 was obtained from Medox Biotech India Pvt Ltd (India). The remaining chemicals and solvents used were of standard analytical grade and HPLC grade, respectively.

### 2.2. Plant Material

*Lawsonia inermis* Linn. (whole plant) was collected in the month of May 2007 from Vellore district (12°55′N, 79°11′E), Tamil Nadu, India, and identified at Botanical Survey of India, Southern Circle, Coimbatore, Tamil Nadu, India. Voucher specimens are maintained at our laboratory for future references (Accession no.: VIT/SBCBE/CCL/07/5/03; May 12, 2007).

### 2.3. Processing and Extraction

Healthy plants were screened for contamination by other species and thoroughly washed. The cleansed plants were freeze dried for 2 months at −80°C in an MDF-U32V V.I.P. Series −86°C Ultra-Low Temperature Freezer (Sanyo Biomedical, IL, USA). The dried plants were powdered for the preparation of extracts. Whole plant powder was serially extracted with methanol and water using Soxhlet apparatus. These crude extracts were concentrated at 40°C under reduced pressure (72 mbar for aqueous extract; 337 mbar for methanolic extract) with a Rotavapor R-215 (BÜCHI Labortechnik AG, Switzerland) to yield dry extracts. Percentage yields of the methanolic and aqueous extracts were, respectively, 19.58% and 10.42% of dry weight. 

### 2.4. Estimation of Antioxidant Potential: Radical Scavenging and Inhibition of Lipid Peroxidation

#### 2.4.1. DPPH Radical Scavenging Activity

The DPPH assay was performed according to the method of Brand-Williams et al. [19] with a few modifications. 2 mL of extract solution (10, 20, 40, 60, 80, and 100 *μ*g mL^−1^) made in methanol was added to 1 mL of DPPH^•^ solution (0.2 mM mL^−1^ methanol) and mixed vigorously. The mixture was incubated in darkness at 20°C for 40 min. Absorbance was measured at 517 nm using a Cary 50 UV-Vis spectrophotometer (Varian, Inc., CA, USA) with methanol as blank. Trolox was used as positive control. The level of percentage scavenging of DPPH^•^ by the extracts was calculated according to the following formula:

(1) $$\text{\%}\;\text{radical}\;\text{scavenging}=\left[\frac{\left(A_{C}-A\right)}{A}\times 100\right],$$

where *A*_*C*_ is the absorbance of the control and *A* is the absorbance of sample. Percentage scavenging was also expressed as Trolox equivalence (in *μ*g mL^−1^).

#### 2.4.2. ABTS Radical Scavenging Activity

ABTS assay was performed according to the protocol of Arnao et al. [20]. Different quantitities (5, 10, 20, and 25 *μ*g) of the phyto-extracts were tested. Absorbance was taken at 734 nm. Percentage scavenging of ABTS^•+^ radical was calculated by a similar formula as used for the calculation of DPPH^•^ scavenging, and also expressed in Trolox equivalence (in *μ*g).

#### 2.4.3. Ferric Reducing Antioxidant Property (FRAP)

FRAP assay was done according to the protocol of Benzie and Strain [21] with some modifications. The stock solutions were 300 mM acetate buffer (3.1 g C_2_H_3_NaO_2_ · 3H_2_O, and 16 mL C_2_H_4_O_2_; pH 3.6), TPTZ solution (10 mM TPTZ in 40 mM HCl), and 20 mM FeCl_3_ · 6H_2_O solution. Working FRAP solution was prepared freshly by mixing 25 mL of acetate buffer, 2.5 mL TPTZ solution, and 2.5 mL of FeCl_3_ · 6H_2_O solution, and then warmed to 37°C before use. 150 *μ*L of individual extract solutions (containing 25, 50, 100, and 200 *μ*g of extracts, resp.) were allowed to react with 2.85 mL of FRAP solution for 30 min in darkness. Absorbance was read at 593 nm. Aqueous solutions of known Fe^2+^ concentrations (FeSO_4_ · 7H_2_O) were used to calibrate the standard curve (Fe^2+^ concentration versus absorbance). Percentage Fe^3+^ scavenging (reduction to Fe^2+^) was calculated by comparison with the standard curve. Percentage scavenging was also evaluated in Trolox equivalence (in *μ*g).

#### 2.4.4. Lipid Peroxidation Inhibition Efficiency

Inhibition efficiency of lipid peroxidation (LPI) was estimated by thiobarbituric acid (TBA) assay [22]. A 6-week-old female Wistar albino rat weighing approximately 150 g was dissected under ethereal anesthesia and its liver was excised. A liver homogenate of 10% (w/v) was prepared in Dulbecco's PBS (Ca^2+^/Mg^2+^-free) (pH 7.4), and centrifuged at 503 g for 15 min to obtain a clear supernatant. Diverse concentrations of each extract were taken in different test tubes and evaporated to dryness at 80°C. 1 mL of 0.15 M potassium chloride and 0.5 mL of the obtained supernatant were added to each tube. Lipid peroxidation was initiated by the addition of 100 *μ*L of 0.2 mM ferric chloride and incubated at 37°C for 30 min. 2 mL of ice-cold 0.25 N hydrochloric acid containing 15% trichloroacetic acid and 0.38% TBA was added to stop the peroxidation reaction, followed by incubation for 1 h at 80°C. The samples were brought down to room temperature and centrifuged at 3144 g for 15 min. Absorbance of the supernatant was measured at 532 nm.

Percentage LPI was calculated by the following formula:

(2) $$\%\;\text{LPI}=\left[\frac{A_{C}-A}{A}\times 100\right],$$

where *A*_*c*_ is the absorbance of the control and *A* is the absorbance of sample. LPI of the extracts were compared with that of BHT and expressed in BHT equivalence. The experiment was performed with the approval of the institutional animal ethical committee (PSGIMSR/27.02.2008) and was in accordance with the “Principles of Laboratory Animal Care” (NIH publication #85–23, revised in 1985) [23].

#### 2.4.5. Test for Inhibition of Cr(VI)-Induced DNA Damage by the Extracts

Efficiency of the extracts as potential DNA protectors against Cr(VI)-induced genotoxicity was tested by treating pBR322 plasmid DNA with CrO_3_ in presence of UV radiation (8000 *μ*W cm^−2^). 1 *μ*L aliquots of pBR322 (200 *μ*g mL^−1^) were taken in four polyethylene microcentrifuge tubes. 50 *μ*g of each extract was separately added to two individual tubes—S_M_ (sample with methanolic extract) and S_A_ (sample with aqueous extract). The remaining two tubes were kept without addition of any extract, and served as controls. 5 *μ*L of 10 *μ*M CrO_3_ was added to both S_M_ and S_A_ and in one of the control tubes (now designated C_Cr_). The other control tube was left without addition of CrO_3_ and was designated as C_0_. C_Cr_, S_M_, and S_A_ tubes were then placed directly on the surface of a UV transilluminator (300 nm) and irradiated for 15 min at room temperature. All DNAs (C_0_, C_Cr_, S_M_, and S_A_) were run on 1% agarose gel (stained with 10 *μ*g *μ*L^−1^ ethidium bromide solution) and photographed on Lourmat Gel Imaging System (Vilbar, France).

#### 2.4.6. Test for Inhibition of Cr(VI)-Induced Cytotoxicity by the Extracts: XTT Assay

Inhibition of Cr(VI)-induced cytotoxicity by the two extracts at various concentrations was tested by the method of XTT-formazan dye formation [24]. MDA-MB-435S cells cultured in L-15 (Leibovitz) cell culture medium (with 10% serum) were seeded (200 *μ*L, 6 × 10^3^ cells/well) in a 96-well plate and allowed to grow for 24 h at 37°C. After incubation, medium was removed from all wells. 200 *μ*L fresh medium was added to the control wells. Cells in each test well were treated with 10 *μ*M CrO_3_ (prepared in medium) along with different extract dosages (0, 125, 250, 500, and 1000 *μ*g). Cells in both control and test wells were re-incubated for 24 h maintaining the same conditions. After the treatment incubation period, medium in each well was substituted by 200 *μ*L of fresh medium followed by the addition of 50 *μ*L of XTT (0.6 mg mL^−1^) containing 25 *μ*M PMS. The plate was further incubated for 4 h in the same conditions. Absorbance was measured at 450 nm (with a 630 nm reference filter) in a Dynex Opsys MR Microplate Reader (Dynex Technologies, VA, USA).

Percentage cytotoxicity was calculated by the following formula:

(3) $$\text{\%}\;\text{cytotoxicity}=\left[\frac{\left(A_{C}-A_{T}\;\right)}{A_{C}}\right]\times 100,$$

where *A*_*C*_ is the mean absorbance of the control wells and *A*_*T*_ is the mean absorbance of test wells with a particular extract dosage.

### 2.5. Analysis of Phenolic Contents

#### 2.5.1. Estimation of Total Phenolic Content

Total phenolic content of the two extracts of *L. inermis* was determined using the Folin-Ciocalteau reagent method [25]. To 50 *μ*L of each extract of different concentrations (125, 250, 500, and 1000 *μ*g), 2.5 mL of Folin-Ciocalteau reagent (1/10 dilution) and 2 mL of 7.5% Na_2_CO_3_ (w/v) were added and mixed well. The blend was incubated at 45°C for 15 min. The absorbances of all samples were measured at 765 nm with Na_2_CO_3_ solution (2 mL of 7.5% Na_2_CO_3_ in 2.55 mL of distilled water) as blank. The results were expressed as GAE (gallic acid equivalence) in *μ*g.

#### 2.5.2. Determination of Phenolic Compounds: HPLC Analysis

HPLC analysis was performed using a Waters 2487 HPLC system consisting of a dual *λ* detector and a Waters 1525 binary pump, and equipped with a Waters Symmetry C18 column (5 *μ*m, 4.6 × 150 mm) with Waters Sentry universal guard column (5 *μ*m, 4.6 × 20 mm) (Waters Corporation, Milford, MA, USA). Phenolic compounds in the aqueous and methanolic extracts of *L. inermis* were analyzed using the reference HPLC method [26]. Gradient elution was performed at 35°C with solution A (50 mM sodium phosphate in 10% methanol; pH 3.3) and solution B (70% methanol) in the following gradient elution program: 0–15 min—100% of Solution A; 15–45 min—70% of Solution A; 45–65 min—65% of Solution A; 65–70 min—60% of Solution A; 70–95 min—50% of Solution A; 95–100 min—0% of Solution A. Flow rate was 1 mL min^−1^ and injection volume was 20 *μ*L. Detection was monitored at diverse wavelengths (around *λ*_max_) for various phenolic compounds, that is, 250 nm for benzoic acids, isoflavones and most anthraquinones; 280 nm for some flavones, flavanones, catechins, theaflavins and some anthraquinones; 320 nm for cinnamic acids, most flavones and chalcones; 370 nm for flavonols; 510 nm for anthocyanins [26].

### 2.6. Statistical Analysis

All analyses were carried out in triplicates. Data were presented as mean ± standard deviation (SD). Statistical analyses were performed by one-way ANOVA. Significant differences between groups were determined at *P* < .05. To evaluate relationships between experimental parameters, results were analyzed for correlation and regression and tested for significance by Student's *t*-test (*P* < .05). MATLAB ver. 7.0 (Natick, MA, USA), SPSS ver. 9.05 (Chicago, IL, USA) and Microsoft Excel 2007 (Roselle, IL, USA) were used for the statistical and graphical evaluations.

## 3. Results

### 3.1. Antioxidant Potential

**Figure 1:**
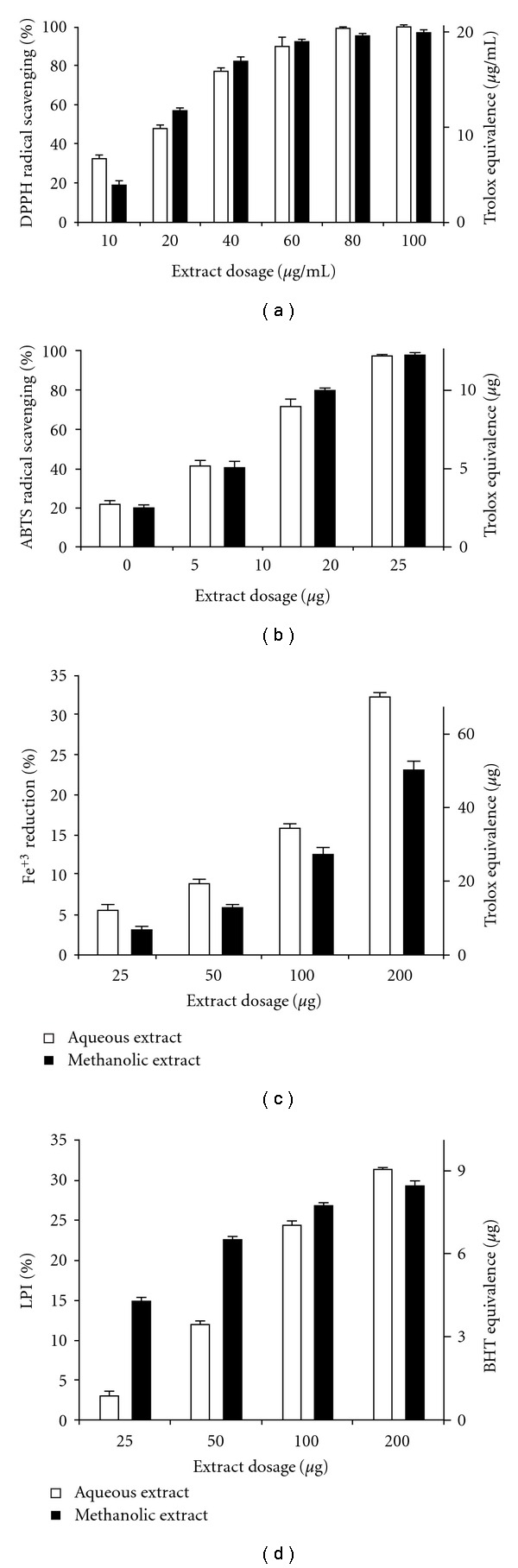
Antioxidant potential of aqueous and methanolic extracts of *L. inermis*. Data expressed are as mean ± SD (*n* = 3, *P* < .05). (a) Percentage DPPH^•^ radical scavenging potential with Trolox equivalence (in *μ*g mL^−1^). (b) Percentage ABTS^•+^ radical scavenging activity with Trolox equivalence (in *μ*g). (c) Percentage Fe^3+^ reducing potential with Trolox equivalence (in *μ*g). (d) Percentage LPI by *L. inermis* extracts with BHT equivalence (in *μ*g).

DPPH^•^ is a stable free radical whose absorbance (*λ*_max_ = 517 nm) decreases when antioxidants donate protons to DPPH^•^ [19]. Quantitative analysis revealed strong DPPH^•^ radical scavenging ability in both the aqueous and methanolic extracts.  Figure 1(a) shows the mean (±SD at *P* < .05) values of percentage DPPH^•^-scavenging for different dosages of the two extracts along with Trolox equivalence.

Figure 1(b) depicts the percentage scavenging of ABTS^•+^ radical by the two extracts and along with Trolox equivalence (in *μ*g). Both extracts showed high antioxidant property, which was in qualitative congruity to the results of the DPPH assay. Quantitatively, however, radical scavenging efficiency was considerably higher in the ABTS assay in comparison to DPPH assay.

Results of the FRAP assay (Figure 1(c)) showed that the aqueous extract was a stronger Fe^3+^-reductant than the methanolic extract. Moreover, both extracts showed considerably lesser antioxidant potential in FRAP in comparison to both DPPH and ABTS assays.

All three radical-scavenging assays showed significant mutual positive correlation at *P* < .05. Coefficient of determination (*R* ^2^) values between DPPH and ABTS, ABTS and FRAP, and DPPH, and FRAP are 0.96, 0.98, and 0.99, respectively.

Intracellular and membrane lipids, when subjected to considerable oxidative stress, lose a hydrogen atom from an unsaturated fatty acyl chain, thus initiating lipid peroxidation which propagates as a chain reaction. This leads to the generation of diverse peroxides and cyclic endoperoxides which consequently form malondialdehyde (MDA). On reacting with TBA, MDA produces a pink chromogen with highest absorbance at 532 nm, thus providing an estimate of LPI [22].  Figure 1(d) shows a dose-dependent inhibition of lipid peroxidation (by both extracts) along with BHT equivalence (in *μ*g).

### 3.2. Inhibition of Cr(VI)-Induced DNA Damage

**Figure 2:**
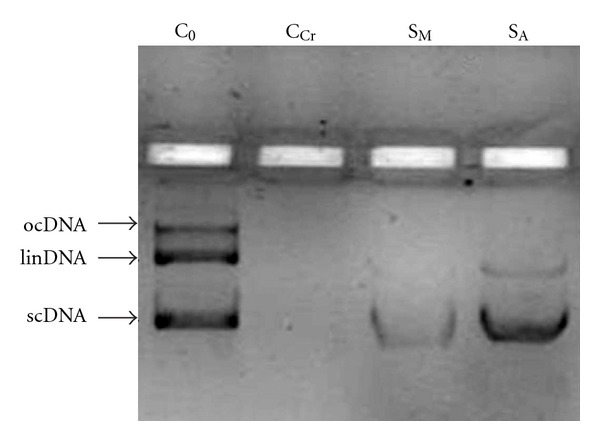
Effect of *L. inermis* extracts (50 *μ*g) on the protection of DNA (plasmid pBR322) against oxidative damage caused by exposure to Cr(VI) (10 *μ*M CrO_3_) and UV radiation. C_0_ = normal without Cr(VI)-UV exposure and extract treatment (control); C_Cr_ = DNA with Cr(VI)-UV exposure and without extract treatment (control); S_M_ = DNA with Cr(VI)-UV exposure, methanolic extract treated; S_A_ = DNA with Cr(VI)-UV exposure, aqueous extract treated; scDNA = supercoiled DNA; ocDNA = open circular DNA; linDNA = linear DNA.

Cr(VI) causes DNA damage involving adducts, breaks, and cross-links and inhibits repair of DNA damage induced by UV [27].  Figure 2 shows the electrophoretic pattern of pBR322 DNA following exposure to Cr(VI)-UV. Normal pBR322 (C_0_) showed three bands on agarose gel electrophoresis. The faster moving band represented the native form of supercoiled circular DNA (scDNA), the slower moving band corresponded to the open circular form (ocDNA), and the intermediate band designates linear DNA (linDNA) [28]. Cr(VI)-UV-exposure caused absolute damage to pBR322 DNA (no bands visible) in C_Cr_. S_A_ demonstrated comparatively robust bands than S_M_. S_A_ showed a banding pattern consisting of a faint linDNA band and a prominent scDNA band, although the band corresponding to ocDNA was lacking. S_M_, on the other hand, showed very faint scDNA band with the ocDNA and linDNA totally obliterated. However, both S_A_ and S_M_ showed some magnitude of DNA protection in comparison to C_Cr_. This suggested that both extracts showed varying degrees of potential in inhibiting DNA damage due to Cr(VI)-UV exposure, the aqueous extract being a better DNA-protector than the methanolic.

### 3.3. Inhibition of Cr(VI)-Induced Cytotoxicity

**Figure 3:**
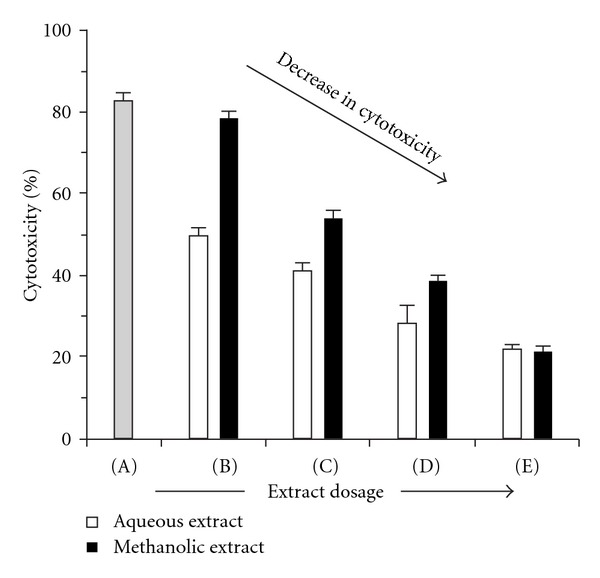
Dosage-dependent inhibition of Cr(VI)-induced cytotoxicity in MDA-MB-435S (human breast carcinoma) cells by aqueous and methanolic extracts of *L. inermis* as estimated by XTT assay. Sample cells were treated with 10 *μ*M Cr(VI) for 24 h with the following extract-dosages: (A): 0 *μ*g; (B): 125 *μ*g; (C): 250 *μ*g; (D): 500 *μ*g; (E): 1000 *μ*g. Control cells (without Cr and/or extract treatment) were used as reference for evaluating percentage cytotoxicity. Values given are as mean ± SD for *n* = 3 samples (*P* < .05).

Live cells metabolically reduce XTT to a soluble product XTT-formazan, which can be estimated spectrophotometrically as a measure of cell viability [24].  Figure 3 shows the protective effects of the aqueous and methanolic extracts on Cr(VI)-induced cytotoxicity in MDA-MB-435S cells as determined by XTT assay. Cells treated with only Cr(VI) were subjected to severely high toxicity. However, cytotoxicity was effectively mitigated in Cr(VI)-treated cells by action of aqueous and methanolic extracts of *L. inermis* in a dosage-dependent pattern.

### 3.4. Phenolic Contents

#### 3.4.1. Estimation of Total Phenolic Content

**Figure 4:**
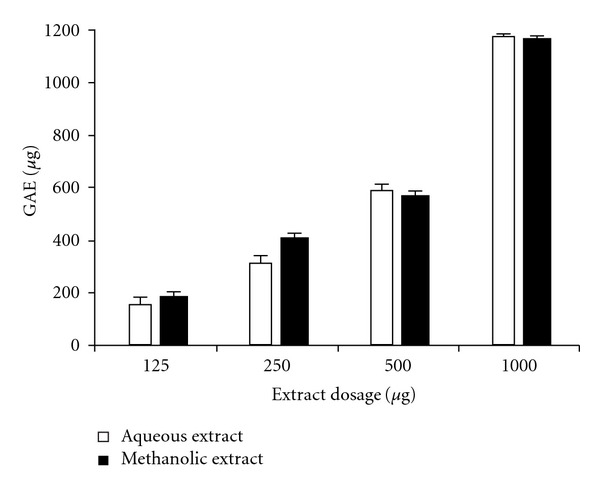
Total phenolic content in varying concentrations of *L. inermis* extracts. Data is given in mean ± SD for *n* = 3 samples (*P* < .05). GAEof the extracts is given in *μ*g.

Phenolic compounds can be defined as a large series of chemical constituents possessing at least one aromatic ring bearing hydroxyl and other subconstituents, including their functional derivatives [29]. Variations in the quantity of total phenolics in the two extracts are presented in Figure 4. Quantitative estimation proved that both extracts have considerably high constitutions of phenolic compounds that increase with extract dosage. Total phenolic content, which was expressed in GAE in *μ*g, demonstrated a much enhanced phenolic composition of both extracts than the gallic acid standard.

**Figure 5:**
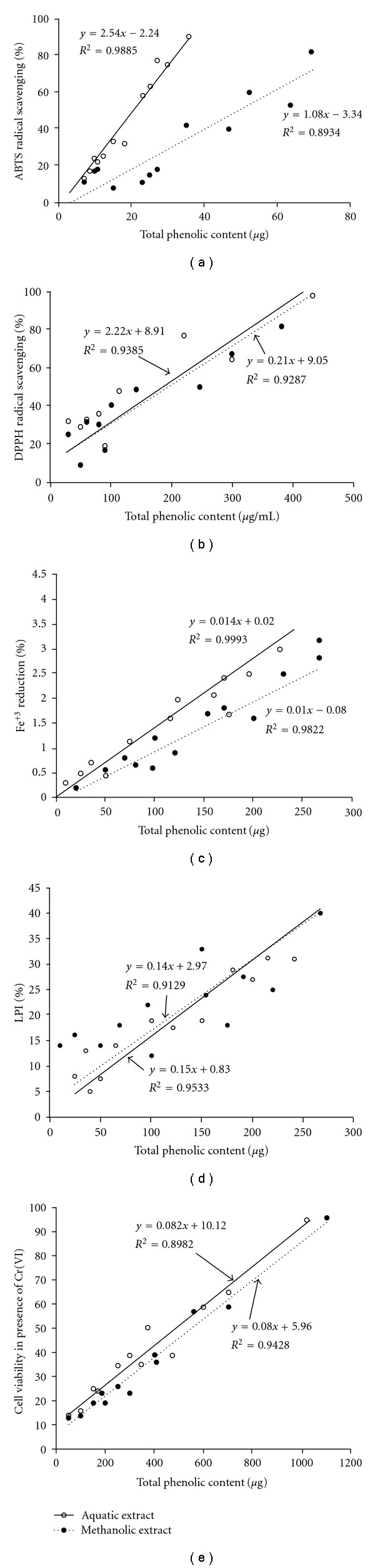
Relationship between total phenolic content of aqueous and methanolic extracts of *L. inermis* and (a) ABTS^•+^ *‏* radical scavenging potential. (b) DPPH^•^ radical scavenging efficiency. (c) Fe^3+^ *‏* reducing potential. (d) LPI. (e) Cell viability against Cr(VI)-induced toxicity. All parameters show strong and significant positive correlation with total phenolic content (at *P* < .05) for both extracts.

Phenolic compounds have been found to encounter heavy metal-induced stress in plants [30]. Further, phenolic compounds are also known for their antioxidant, anti-mutagenic and anti-tumor activities [31]. To check whether the phenolic content of the two extracts can be accredited for their antioxidant potential and their property to inhibit Cr(VI)-induced cytotoxicity, correlation and regression analyses were performed. Total phenolic content of both extracts showed significant and strong positive correlation (*P* < .05) with free radical scavenging efficiency (DPPH, ABTS and FRAP assays), potential of LPI (TBA assay) and property of inhibition of Cr(VI)-induced oxidative cytotoxicity (Figure 5).

#### 3.4.2. Determination of Phenolic Compounds: HPLC Analysis

Due to the diversity and complexity of natural phenolic compounds, it is hard to characterize every compound and elucidate its structure [13]. The major types of phenolic compounds in the two extracts of *L. inermis* were determined by HPLC analysis. A library of the analytical characteristics (*λ*_max_, retention time, determining *λ*, slope and limit calibration) of more than 100 phenolic standards established by Sakakibara et al. [26] was used as the reference for compound identification.  Table 1 shows the major phenolic compounds in the two extracts.

## 4. Discussion

Aqueous and methanolic extracts of *L. inermis* showed considerable antioxidant potential in all the analytical studies. The results of the DPPH, ABTS and FRAP assays were in congruity with those previously reported [13], and additionally, proved a dosage-dependent increase in antioxidant potential over different ranges with distinct extract-specific efficiencies. The differential scavenging activities of the extracts against DPPH^•^, ABTS^•+^, and Fe^3+^ radicals may be referred to the different mechanisms of the radical-antioxidant reactions in these assays. The stoichiometry of reactions between the antioxidant compounds in the extracts and the DPPH^•^, ABTS^•+^, and Fe^3+^ radicals is distinctively dissimilar, which may be inferred as a reason for the difference in scavenging potential. The diversity in radical scavenging shown in these assays may also be due to factors like stereoselectivity of the radicals or the differential solubility of the extracts [32] in the three testing systems. Therefore, the considerable difference in antioxidant efficiency for both extracts among the three models is justified.

A previous study [33] reported that aqueous solution of commercially available *L. inermis* powder did not show any prominent inhibition of lipid peroxidation (i.e., no decrease in the level of MDA in test samples in comparison to an untreated control group) in the liver cells of female Swiss albino rats. The results of the present study, however, showed considerable difference in results, and found both extracts efficient in this precinct. Treatment by both extracts showed a significant (*P* < .05) dosage-dependent increase in the capacity to inhibit lipid peroxidation in the rat liver cells. The reason for this difference in results might be attributed to the presence of toxic substances like para-phenylenediamine (PPD), nickel, cobalt, and so forth. in commercially available *L. inermis* powder [34] which was used for the previous study [33]; and hence it might not have inhibited lipid peroxidation.

pBR322 DNA was protected by both extracts against 10 *μ*M Cr(VI) in presence of UV radiation. Although such a protective potential is extensively dependent on the dosage of both Cr(VI) and the extracts, it is evident that both aqueous and methanolic extracts demonstrated variable magnitudes of DNA protection against Cr(VI)-induced oxidative stress which completely damaged DNA in absence of any extract (i.e., in C_Cr_).

A steady decline was noted in the magnitude of Cr(VI)-induced cytotoxicity in MDA-MB-435S cells with increasing dosage of the extracts in the XTT assay. The aqueous extract proved to be a considerably better cyto-protective agent in comparison to the methanolic extract at 125, 250, and 500 *μ*g dosages. At 1000 *μ*g, however, both extracts showed almost identical levels of protection to the cells, with the aqueous extract minutely ahead of its counterpart. ROS causes oxidative damage to genomic DNA, mtDNA, lipids (lipid peroxidation) and proteins, and leads to cellular dysfunction and/or cell loss through energy deficit and apoptosis [10, 35]. Cr(VI), being an efficient generator of ROS [9], thereby causes considerable cytotoxicity to cells through free radical-mediated oxidative cascades [11, 12]. Both aqueous and methanolic extracts of *L. inermis* have demonstrated effective potential in free radical scavenging and lipid peroxidation inhibition, by virtue of which they may have been able to counter the oxidative stress generated in MDA-MB-435S cells by Cr(VI).

Phenolic content of both extracts showed significant correlation with free radical scavenging, LPI and cytoprotective potential against Cr(VI)-induced toxicity. HPLC analysis revealed presence of a variety of phenolic compounds in both extracts which might have been responsible for their effective therapeutic potentials as reported by this study. However, it is interesting to note that the diversity of phenolic compounds in the aqueous extract is colossally more in comparison to that of the methanolic extract.

**Figure 6:**
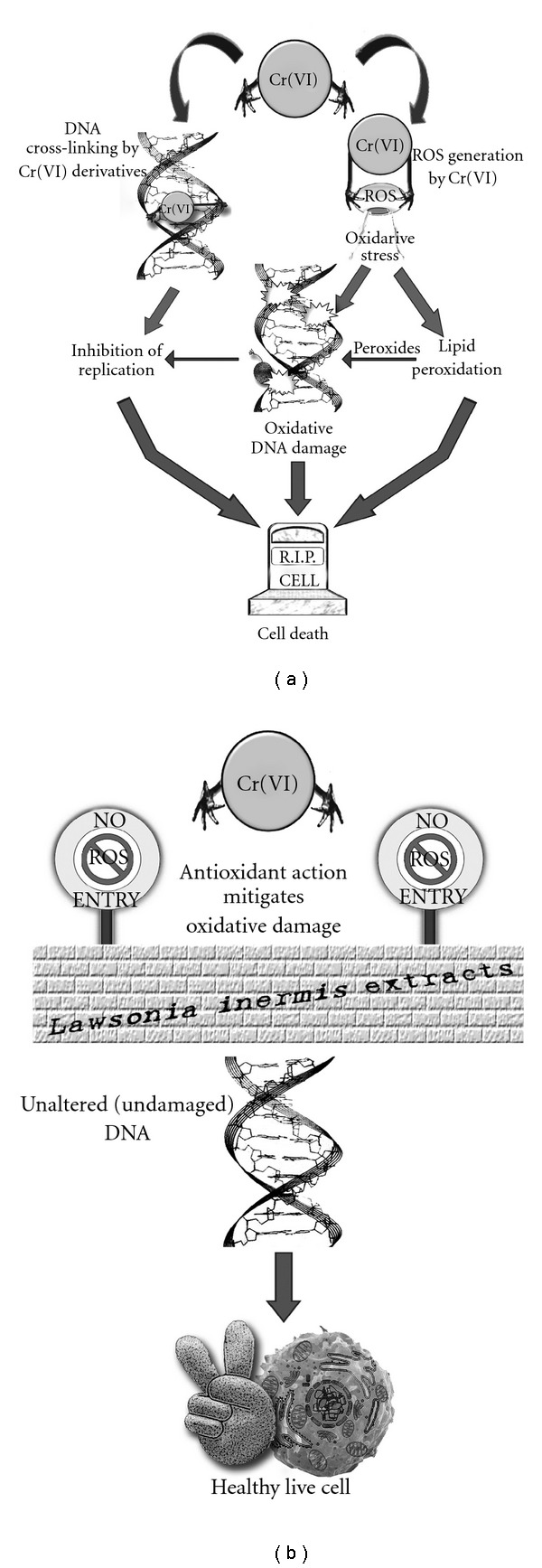
Animated hypothetical diagram representing the mode of action of *L. inermis* extracts against Cr(VI)-induced cytotoxicity. (a) Cr(VI) cross-links DNA and causes oxidative damage to DNA and lipids, consequently leading to cell death. (b) *L. inermis* extracts provide a barrier against Cr(VI) toxicity cascade by virtue of antioxidant efficacies, thereby ensuring cell viability.

In conclusion, it can be inferred that aqueous and methanolic extracts of *L. inermis* have high antioxidant potential (by virtue of their diverse phenolic constituents) which simultaneously inhibits Cr(VI)-induced oxidative toxicity to MDA-MB-435S cells and pBR322 DNA. Hence, the tested extracts inhibit the oxidative damage pathway induced by Cr(VI), and thereby prevent cell death (Figure 6). The plant can serve as a prospective source of natural phenolics and other metabolites which could prove to be precursors for designing effective drugs against heavy metal toxicity.

## Figures and Tables

**Table 1:** Major phenolic compounds present in aqueous and methanolic extracts of *L. inermis* as determined by HPLC.

Phenolic compounds	*Lawsonia inermis* aqueous extract	*Lawsonia inermis* methanolic extract
Simple polyphenols		
Chlorogenic acid (caffeoylquinic acid)	+	−
Ferulic acid	+	−
Gallic acid	+	+
Isoferulic acid	+	−
*m*-coumaric acid	+	−
*o*-coumaric acid	+	−
*p*-hydroxybenzoic acid	−	+
Flavonoids (flavones, flavonols, and flavanones)	
7, 4′-dihydroxyflavone	+	−
Apigenin	+	−
Flavone	+	−
Flavonol	+	−
Kaempferol	+	−
Luteolin	+	−
Luteolin-7-O-glucoside	+	−
Myricetin	+	−
Naringenin-7-O-rutinoside	−	+
Quercetin	+	−
Vitexin-2′-O-rhamnoside	+	−
Catechins		
(+)—catechin	+	−
(−)—catechin gallate	+	−
(−)—epicatechin gallate	+	−
Chalcones		
Butein	+	−
Chalcone	+	−
Phloretin	+	−
Anthocyanins		
Cyanidin	+	−
Cyanidin-3-*O*-rutinoside	+	−
Pelargonidin	+	−
